# *In Vivo* Solid-Phase Microextraction and Applications in Environmental Sciences

**DOI:** 10.1021/acsenvironau.1c00024

**Published:** 2021-10-24

**Authors:** Miao Yu, Anna Roszkowska, Janusz Pawliszyn

**Affiliations:** † Department of Environmental Medicine and Public Health, Icahn School of Medicine at Mount Sinai, New York, New York 10029, United States; ‡ Department of Pharmaceutical Chemistry, Medical University of Gdansk, Gdansk 80-416, Poland; § Department of Chemistry, University of Waterloo, Waterloo, ON N2L 3G1, Canada

**Keywords:** SPME, *in vivo* sampling, environment, toxicology, contaminants, calibration, bioanalysis

## Abstract

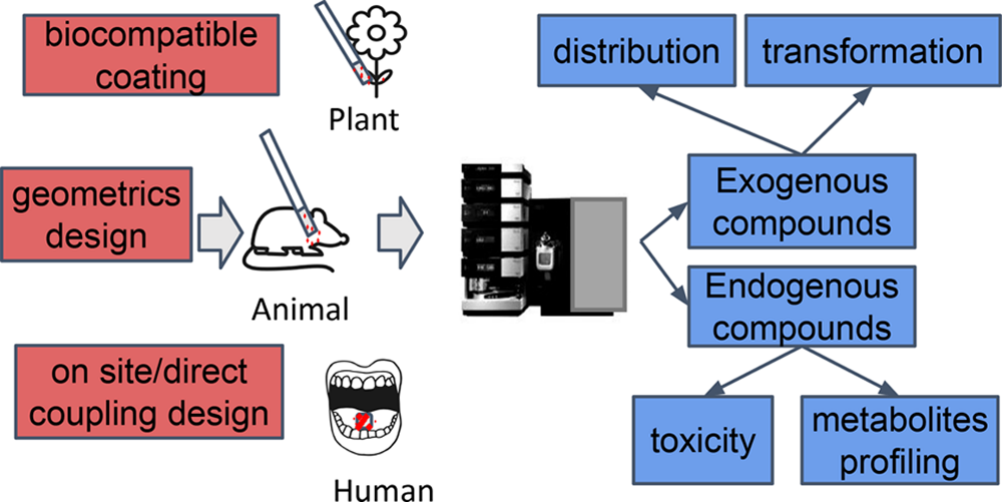

Solid-phase microextraction (SPME) is a well-established sample-preparation technique for environmental studies. The application of SPME has extended from the headspace extraction of volatile compounds to the capture of active components in living organisms via the direct immersion of SPME probes into the tissue (*in vivo* SPME). The development of biocompatible coatings and the availability of different calibration approaches enable the *in vivo* sampling of exogenous and endogenous compounds from the living plants and animals without the need for tissue collection. In addition, new geometries such as thin-film coatings, needle-trap devices, recession needles, coated tips, and blades have increased the sensitivity and robustness of *in vivo* sampling. In this paper, we detail the fundamentals of *in vivo* SPME, including the various extraction modes, coating geometries, calibration methods, and data analysis methods that are commonly employed. We also discuss recent applications of *in vivo* SPME in environmental studies and in the analysis of pollutants in plant and animal tissues, as well as in human saliva, breath, and skin analysis. As we show, *in vivo* SPME has tremendous potential for the targeted and untargeted screening of small molecules in living organisms for environmental monitoring applications.

## 1 Introduction

The analysis of contaminants in environmental samples is the first step in evaluating the negative impact of such compounds in an ecosystem and the living organisms within it. *In vivo* studies are highly useful for such studies, as they can reveal the temporal and longitudinal distribution of environmental contaminants and their effects on plants, animals, microorganisms, and/or humans.^1^ In environmental science, *in vivo* analysis can be used to investigate the distribution patterns^2^ and transformation products of exogenous compounds^3^ as well as their interactions with endogenous compounds that occurring naturally in the living system.^4^*In vivo* studies enable environmental scientists to monitor certain environmental processes of specific compounds in real time, thus allowing them to collect biological information about living systems at the molecular (biochemical) level. However, such studies require a feasible analytical method that facilitates throughput/efficient isolation and quenching of exogenous and endogenous compounds of interest along with precise analysis.

Solid-phase microextraction (SPME) was introduced in 1989 as a fast, simple, and green sample-preparation technique that can be used with a wide range of samples.^5^ SPME combines sampling, cleanup, and preconcentration into a single step and can be coupled with modern instruments, such as gas chromatography–mass spectrometry (GC-MS)^6^ and liquid chromatography–mass spectrometry (LC-MS).^7^ Recently, researchers have enabled fast and high-throughput analysis in pharmaceutical studies via the direct coupling of SPME devices to MS.^8^ SPME is a nonexhaustive extraction method, which means that it only extracts a free fraction of the analyte from the sample matrix. This feature enables the measurement and discussion of the bioavailability of various environmental contaminants.^9^

SPME was initially introduced as novel extraction strategy in environmental studies, but it has since become a leading method in this scientific field. Currently, SPME—and headspace-solid-phase microextraction (HS-SPME) in particular—is widely employed in analyses of volatile organic compounds (VOCs) and semivolatile organic compounds (SVOCs).^10−12^ In addition, the availability of different extraction phases makes it possible to use SPME devices to extract pollutants with a range of different physicochemical properties, such as pharmaceutical and personal care products (PPCPs), pesticides, and metal organic compounds, among others.^13−15^ It is generally thought that the free concentration of a given compound is a stronger indication of its bioavailability compared to its total concentration.^16^ By extracting a small portion of the free fraction of analytes (negligible depletion), SPME enables the direct measurement of the free concentration of certain contaminants in environmental samples. Specifically, SPME can be used to monitor dynamic biological processes in living systems, and it also makes it possible to perform repeated extractions from a single spot on a given sample. Furthermore, SPME has also been optimized for on-site environmental analysis with microinstruments, such as field-water sampling.^17^ Other extraction methods such as liquid–liquid extraction (LLE) or solid-phase extraction (SPE) can also be applied in *in vivo* or *ex vivo* studies; however, those techniques could not be directly used for *in vivo* sampling performed without the collection of tissue biopsy or biofluid. In addition, other *in vivo* sampling methods such as microdialysis (MD) show a different coverage of metabolites than *in vivo* SPME does, and this is because MD primarily facilitates the extraction of polar species while SPME covers semihydrophobic and hydrophobic compounds as reported for the sampling of brain tissue.^18^

SPME has been applied in *in vivo* environmental studies to monitor changes in small endogenous molecules as well as to directly monitor pollutants in living organisms.^19^ Additionally, SPME is a nonexhaustive and nonlethal sampling method, which has enabled its use for measuring the free concentration of drugs in solid tissue, both via a series of laboratory experiments and *in silico* using a mathematical model developed in COMSOL Multiphysics.^20^ The *in vivo* application of SPME in environmental studies allows researchers to dynamically analyze the distribution, accumulation, and depreciation of single or multiple molecules, as it enables the repeated sampling of the same tissue in a subject.^8^*In vivo* SPME sampling can also capture short-lived and unstable metabolites by stabilizing highly reactive small molecules, which may undergo degradation during sample handling and storage in typical *ex vivo* studies.^21^ This is a significant feature, as such metabolites may provide additional information about metabolic changes, for instance, in exposome-wide association studies (EWAS).^22^

Whereas previous reviews have mainly focused on the development of the *in vivo* SPME technique,^1,8,23,24^ this review examines how *in vivo* SPME has been applied in environmental studies over the past 5 years. We begin by providing a brief overview of the fundamentals of *in vivo* SPME and some related techniques before moving on to a more detailed discussion of its most important aspects, namely, the development of *in vivo* SPME devices, calibration methods, and data analysis. Next, we conduct a review of the main applications of this technique in environmental studies monitoring plant, animal, and human systems. Finally, this paper concludes by considering future possibilities for SPME and its potential in environmental studies.

## 2 Fundamentals of *In Vivo* SPME

### 2.1 *In Vivo* SPME and Related Techniques

Small molecules in environmental samples can be distributed or released into the sample’s gas, liquid, and solid phase. HS-SPME is a commonly used approach for capturing the free concentration of pollutants in the gaseous phase. In this extraction mode, an SPME fiber is exposed to the air phase above samples to extract volatilized compounds. In contrast, needle-trap devices (NTD) enable exhaustive extraction by using small needles containing a packed sorbent bed to trap both fluid-borne analytes and particles. Both HS-SPME fibers and NTDs can be directly coupled to GC injection systems, wherein the analytes adsorbed from the air phase are released for instrumental analysis. In this case, NTDs and SPME fibers can be used in concert to detect both the free and total concentrations of pollutants in the air phase, thus providing a comprehensive assessment of the risks associated with certain environmental processes.^25^ Moreover, cold fiber SPME (CF-SPME)—which entails heating the sample matrix and cooling the fiber coating simultaneously—can be used to increase sensitivity to VOCs released from soil and sediment samples.^26,27^ This technique implies the need for a system to control the SPME fiber’s temperature in order to capture trace level molecules released by a living system. Furthermore, the use of SPME-Arrows can offer improved sensitivity and mechanical robustness for air-phase extraction, as they contain larger volumes of sorbent than standard HS-SPME fibers.^28^

**Figure 1:**
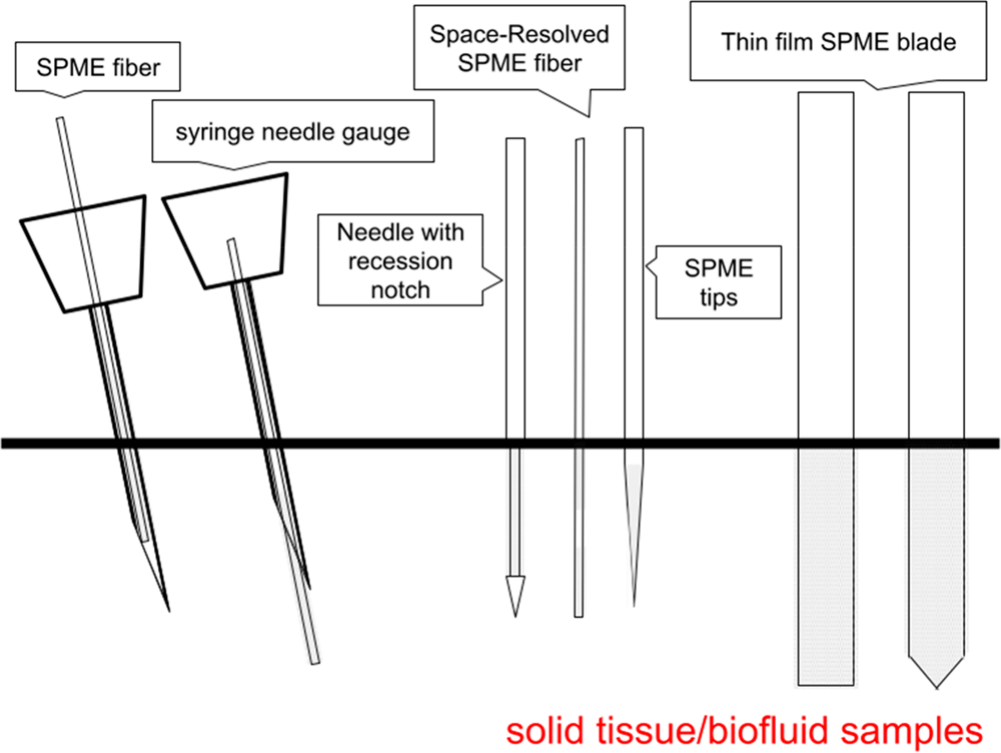
Common SPME device design with the use of biocompatible coatings used in *in vivo* studies. From the left to right, the devices are SPME fiber with a syringe needle gauge to precisely deliver fiber into the tissue/biofluid of the living system; needle with recession notch to avoid mechanical damage of the fiber; space-resolved SPME fiber to extract metabolites from different layers of a semisolid/solid tissues; SPME tips to extract from samples with small size; and thin film SPME blade with larger surface area.

On the other hand, direct immersion SPME (DI-SPME) is the major extraction mode for liquid, solid, and semisolid environmental samples. In DI-SPME, SPME devices coated with an extraction phase are immersed into the samples for a fixed time; the analytes are then desorbed from the extraction phase by directly coupling the device to mass spectrometry (MS) or by applying an optimized solvent, which is subsequently subjected to LC-MS analysis. The application scope of DI-SPME has gradually broadened with the development of new SPME device geometries.^29^ For instance, in-tube SPME has been applied in environmental studies for its ability to directly measure nonvolatile compounds in aqueous matrices.^30^ Needles with a recession notch coated with an extraction phase can protect against mechanical damage and have been applied in untargeted analyses using living fish.^31^ Space-resolved SPME with discontinued coatings can be used to perform simultaneous extractions from different tissue *in vivo*.^32^ Coated tips and minitips, which were developed based on the a coated tip apex (1 mm), allow the extraction of small molecules from limited sample amounts (<10uL), such as blood from mice.^33,34^ Such designs also allow direct coupling with MS for high-throughput analysis and can be applied for nontarget analysis—features which are highly beneficial in exposome studies. In SPME, larger surface areas will result in improved extraction efficiency and sensitivity.^35^ Thin-film SPME (TFME) offers a larger surface area to extraction phase volume ratio, which means that more of the device is coming into contact with the sample. Depending on the research objectives, the TFME membrane coatings can be modified for gas chromatography or liquid chromatography. In addition, TFME and CF-SPME can also be combined to further enhance method sensitivity for specific applications, such as measuring fragrance compounds in the air.^36^ Coated blade spray (CBS) is another geometry that allows sample preparation to be directly connected to MS, and it has also been used in environmental analyses.^37^ Furthermore, the analysis of nonvolatile pollutants via GC has been enabled by the development of an on-fiber derivatization technique that combines the derivatization reaction and extraction steps during SPME.^38^ Notably, simulation-based geometry optimization can also be used to design new SPME devices based on the aims of the research and the sample matrix.^39^Figure 1 shows the common SPME device design for *in vivo* studies.

SPME can also be optimized for *in vivo* sampling via coating optimization. For *in vivo* studies, the extraction phase coating should be safe and biocompatible with the organism being studied. Since a pure PDMS extraction phase possesses antifouling properties, PDMS-coated SPME fibers, such as PDMS/DVB/PDMS, are able to provide stable extraction performance after over 100 extraction/desorption cycles.^40^ SPME coatings that use polytetrafluoroethylene amorphous fluoroplastics (PTFE AF 2400) as a particle binder—which can hold hydrophilic–lipophilic balance (HLB) adsorptive particles covering compounds with a broad range of hydrophobicities—have also frequently used for *in vivo* analysis.^41^ Other biocompatible coatings, such as C_18_, mixed C_18_ (mixed-mode) and cation-exchange particles, polydopamine (PDA), polymeric ionic liquids (PILs), and polyacrylonitrile (PAN), are also suitable for *in vivo* studies.^42^ Furthermore, the introduction of nanomaterials and electrosorption makes it possible to shorten the extraction process with *in vivo* SPME fibers to around 1 min.^43^ Besides assembling multiple SPME fibers with different coatings as a “swab” for *in vivo* saliva analysis, the chemical coverage of these coatings can be extended to cover different environmental pollutants or endogenous metabolites.^44^ In addition, coatings composed of modern components, such as nanomaterials, metal–organic frameworks (MOFs), and molecularly imprinted polymer (MIPs), have enhanced the durability, sensitivity, and specificity of *in vivo* SPME studies.^8^

On-site biomonitoring and high-throughput analysis have also benefited from constant developments and improvements to SPME. For instance, the development of the microfluidic open interface (MOI)^45^ and CBS^46,47^ have increased the throughput of the regular analytical workflow. Furthermore, a mechanically robust SPME sampler composed of six stainless-steel bolts coated with a layer of HLB/PAN particles was designed for the on-site extraction of untargeted pollutants in environmental waters.^48^ Moreover, the development of drone-based TF-SPME water sampling^49^ and USB-powered CBS^50^ with a transportable MS device has also extended the scope of on-site analysis and could be used in future *in vivo* environmental studies.

### 2.2 Calibration

**Figure 2:**
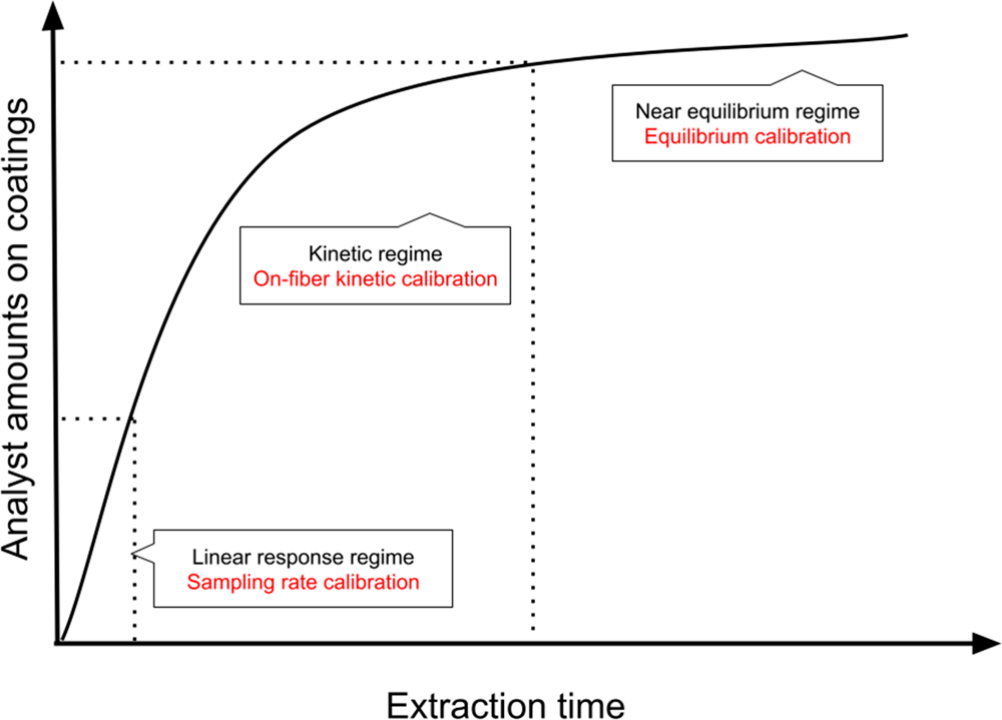
Typical extraction time profile of SPME technique and corresponding calibration methods.

The quantitative analysis of compounds extracted via SPME differs from typical exhaustive extraction analysis. As such, various calibration methods have been developed to quantify the concentrations of target analytes extracted from biosamples using SPME extraction phases.^51^ In SPME, the microextraction of a given compound can be performed after equilibrium has been achieved between the concentration of the target compound in the sample matrix and the extraction phase (SPME coating). The amount of the compound captured by the extraction phase can be expressed as
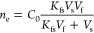
1where *n*_e_ is the amount extracted, *C*_0_ is the initial concentration of the target analyte in the sample, *V*_s_ is the sample volume, *V*_f_ is the volume of extraction phase, and *K*_fs_ is the distribution coefficient of the analyte between the extraction phase and the sample matrix. However, in most SPME applications, the volume of the sample matrix is much larger than the volume of the SPME coating (*V*_s_ ≫ *K*_fs_*V*_f_); thus, eq 1 can be rewritten as
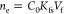
2A typical extraction time profile can be shown in Figure 2. Under equilibrium conditions, calibration is independent of hydrodynamic variables, and eq 2 can be used for quantitative analysis. To conduct a targeted *in vivo* SPME study, it is recommended to generate the compound’s extraction time profile in advance to select the appropriate calibration methods. External mass transfer would impact the calibration approach, especially when the sample matrix is complex. For example, the tissue of the living organism would contain both the solid phase and liquid phase. The *K*_fs_ would change due to the external mass transfer among two different phases. In this case, loading internal standards on the fiber could be a convenient option for method calibration. More diffusion-based calibration methods can be found in previous work.^52^

However, other calibration methods can be used for compounds that require longer times to reach equilibrium, for example, on-fiber kinetic calibration and sampling rate calibration.^53,54^ Such kinetic calibration models require isotope-labeled standards to be preloaded onto the fiber. During microextraction, the labeled standards are desorbed into the sample matrix and the analytes are extracted into the SPME coatings, and the free concentration of analytes is then calculated based on the amount of remaining labeled standards and the extracted compounds. In this case, the losing percentage of isotope labeled standards on the fiber will be equal to the increasing percentage of analyst on the fiber:
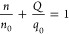
3Researchers can determine *Q* as the amount of remaining isotope labeled standards on the fiber after a fixed time, *q*_0_ as the original isotope labeled standard loading on the fiber, and *n* as the amount of analyte on the fiber. Then the amounts in the samples can be calculated by eq 3. This calibration method improves the accuracy and precision of SPME analysis, while also accounting for the influence of certain environmental factors, such as temperature. However, such an approach may not be feasible for some *in vivo* studies due to the introduction of exogenous compounds (*i.e..*, internal standard) into the living tissue or biofluids.

Sampling rate is another calibration method that can be used in *in vivo* studies. This method is based on the pre-equilibrium stage of a fast microextraction, wherein the amounts of extracted analytes follow linear response patterns (see Figure 2). In this case, when the extraction time is fixed, the extracted amount will be proportional to the concentration of the analyte in the sample matrix:
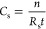
4*C*_s_ is the concentration in the samples, *n* is the detected amounts on the fiber, *t* is the sampling time, and *R*_s_ is the sampling rate of a certain analyte, which can be determined in advance by a spike-in experiment. Then the concentration of analyte in the samples can be calculated by eq 4. In contrast to the equilibrium extraction approach, the sampling rate method is usually applied in contexts requiring fast on-site analysis after the sampling rate of targeted compounds has been measured and pre-established in the laboratory conditions.^55^

Another advancement in *in vivo* SPME technology relates to the calibration method used for semisolid tissue sampling. Specifically, Jiang *et al.* have recently proposed a new theoretical model for the kinetic extraction process in semisolid tissues.^56^ According to their model, since the desorption time constant has a linear relationship with a certain power of molar volume, this relationship can be used to reduce the use of internal standards for the *in vivo* SPME analysis of environmental pollution in semisolid tissues.

*In vivo* environmental studies have been undertaken in an attempt to discover unstable or unknown compounds, such as transformation products or intermediate and transient metabolites of biological processes. However, a calibration method for such unknown compounds remains an analytical challenge without the addition of standards or knowledge of the analytes’ chemical structures. Therefore, untargeted MS-based analysis usually uses features (identical peaks across samples), numbers, identified compounds, or internal standards to compare different extraction methods.^57^ If internal standards are selected for the *in vivo* SPME workflow, their properties, such as cLogP, should be within the range of regular environmental pollutants.^58^

### 2.3 *In Vivo* SPME Workflow

**Figure 3:**
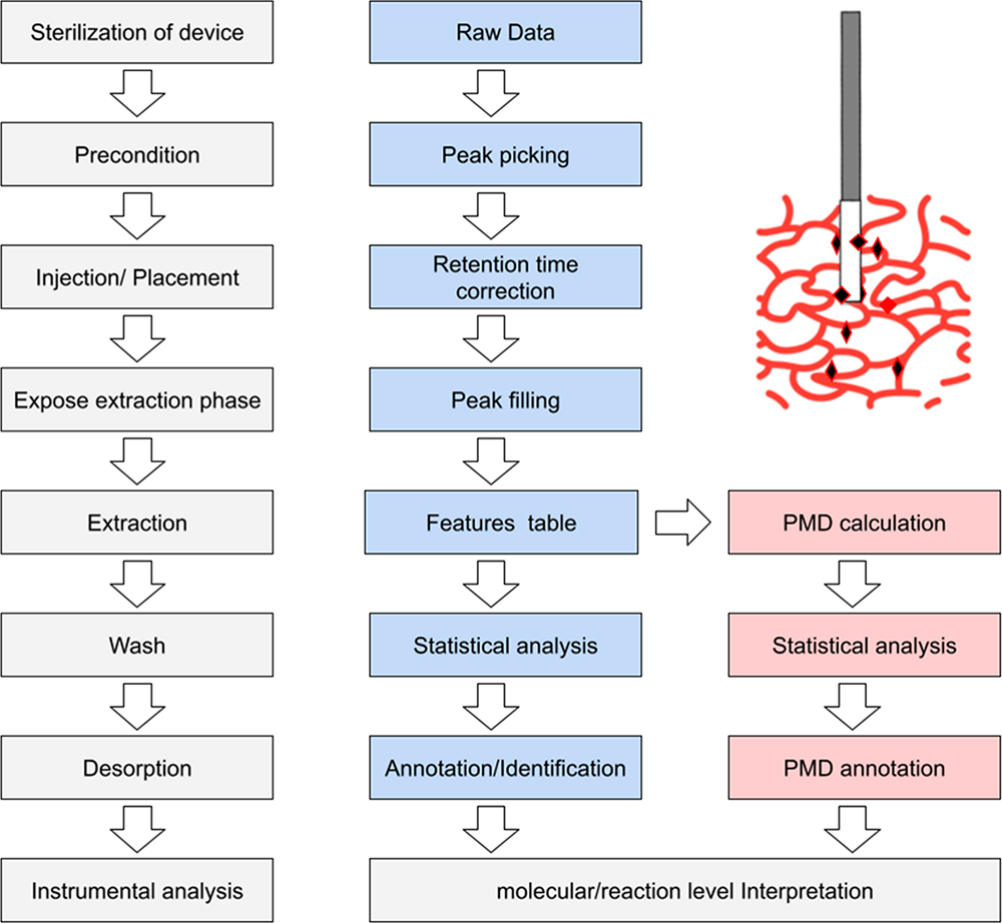
Typical *in vivo* SPME sampling workflow (left) and data analysis workflow for *in vivo* untargeted data in LC/GC-MS-based studies (right).

A typical environmental sample-preparation protocol includes sample collection, homogenization, extraction of small molecules, and cleanup prior to instrumental analysis.^59^ However, for *in vivo* studies, the sample-preparation protocol should minimally impact both the regular functioning of the living organism being studied and the ability to perform dynamic sampling. As shown in Figure 3, sterilizing the SPME devices should be the first step in the *in vivo* SPME analytical protocol, as this helps to ensure the living system under investigation is protected from contamination. For *in vivo* SPME sampling devices, the solid extraction phase should be introduced into the living tissue using a hypodermic needle or directly placed in the biomatrix for the extraction. After a fixed time, the extraction phase is removed from the living system and the analytes are desorbed into an appropriate mixture of solvents, which is followed by instrumental analysis. *In vivo* SPME should use biocompatible coatings, and several parameters affecting extraction efficiency, including precondition time, extraction time, and desorption time, should be optimized for certain analytes. Moreover, SPME can be directly coupled to MS to provide close-to-real-time information about metabolic changes, as well as their levels, in the analyzed matrices.

Depending on the research objective, *in vivo* SPME studies fall into one of two major classes: (1) investigations of the content and behavior of environmental pollutants and (2) analyses of how environmental contaminants impact the functioning of living organisms (metabolomic profiling). Both classes involve the targeted and untargeted analysis of exogenous and endogenous compounds, and *in vivo* SPME devices are capable of extracting these molecules at the same time. In the first approach, the environmental behavior of exogenous compounds includes the targeted analysis of certain compounds and their known metabolites as well as not yet discovered products of their transformations. In the second approach, the environmental analysis within the living organisms comprises the extraction of known endogenous compounds as well as unknown metabolites. The *in vivo* SPME workflow can be optimized for certain compounds during targeted analysis; however, advanced data analysis is particularly important in untargeted analyses, as it facilitates the extraction of reliable *in vivo* bioinformation.

One of the issues of *in vivo* SPME sampling is the coverage of analytes by SPME coatings. *In vivo* study usually needs to calibrate multiple compounds such as exposure compounds, their biotransformation products and/or various metabolites in the environmental analysis. Those analytes show different *K*_fs_ and a fixed time extraction process will capture compounds in different stages of reaching the equilibrium (see Figure 2). One of proposed solutions is to use long extraction and desorption times to make sure that most of the detectable compounds can reach equilibrium on the fiber. On the other hand, coatings with a broader coverage of analytes, such as HLB adsorptive particles or mixed-mode (C18 and cation exchange) particles can be used in *in vivo* studies to capture compounds with different physicochemical properties.^44^ Alternatively, researchers can use multiple fibers with different coatings to extract compounds with different polarities and to investigate ongoing biochemical processes in the living system and, later on, as a part of method development, to optimize the extraction parameters to extract compounds with similar physicochemical properties.^44^ When the *in vivo* SPME study is designed to find the differences in the level of specific metabolites (biomarkers) among studied groups, a relative quantification based on the response of known and unknown compounds can be performed without standards. However, a calibration based on compounds with similar physicochemical properties or synthesized standards should be performed to validate the findings after metabolomics data analysis. Such validation is typically missing in *in vivo* studies due to either the availability of standards or the complexity of experiments. In most *in vivo* studies, targeted compounds for method development are used, which however limits its application for other compounds captured by SPME fibers. In this case, *in vivo* analytical method development should consider the simultaneous calibration of compounds with different physicochemical properties such as polarity or log *K*_ow_.

Another important consideration in the *in vivo* SPME analytical workflow is the impact of sample storage on the biochemical profile and composition of the analytes. For example, *ex vivo* SPME analysis of fish muscle samples that had been stored for 1 year revealed a 10-fold decrease in the number of detected molecular features compared to *in vivo* SPME performed on living fish.^21^ Thus, *in vivo* SPME extracts should be analyzed in real time to avoid the detrimental impacts of storage. As confirmed in a recent study, instrumental analysis immediately following *in vivo* SPME sampling and a reverse time series experimental design should be the preferred approaches for the metabolomic profiling of unstable compounds, while storing SPME fibers or the desorption solution for 1 month will not affect the metabolic profile. At the reaction level, metabolites involved in homologous series with butylation reactions were shown to be the most stable during storage.^60^

### 2.4 Data Analysis for *In Vivo* SPME

For targeted analyses, the data analysis procedure for *in vivo* SPME and regular SPME is nearly the same. However, the workflow for the two types of SPME analysis differs significantly for untargeted analysis, such as LC-MS-based analysis. At present, untargeted analysis, or nontargeted analysis, is the preferred method for screening harmful compounds or finding linkages between environmental exposure and endogenous metabolites.^61^ As shown in Figure 3, untargeted data should be processed to generate a features table via peak picking, retention time correction, and peak filling of missing value during the peak picking. The features table can then be used to perform statistical analysis such as differential analysis to screen features of interests of certain environmental processes. After statistical analysis, the annotation or identification process can be performed to assign names, structures, or molecular formulas to the features for biological interpretation at the molecular level. This type of data analysis workflow usually requires more time than sample analysis.

The annotation of small molecules from living samples remains a challenge for *in vivo* studies.^62^ Findings have demonstrated that *in vivo* SPME is able to capture active compounds that might disappear after even 1 day of storage at −80 °C;^60^ however, such short-lived compounds may never be reported or fully investigated for biological functions. In this case, *in vivo* study usually only reports a few known compounds with available standards, some tentative compounds with database entries, and a lot of totally unknown features from instruments. Biological functional discussions of *in vivo* studies exclusively focus on known compounds, which omits any discussion of their origins and relations with other compounds.^19^

To address this issue, reaction/structure directed analysis was developed for *in vivo* SPME-based studies.^63^ Under this approach, the mass-to-charge ratios of unknown compounds are coupled with fragmental ions for identification.^64^ Furthermore, the distances between the mass-to-charge ratios may reveal certain types of reactions or structures, while the data mining of known reactions can serve to validate such phenomena.^65^ For instance, a paired-mass distance (PMD) of 2.02 Da is always associated with a double-bond broken process. The extension of reaction/structure directed analysis, namely, PMD-based reactomics, further provides the qualification and quantification methods for PMD analysis.^65^ In this case, evaluating changes in certain PMDs enables researchers to determine the reaction level changes within the sample^60,66,67^ and skip the annotation of specific compounds. In addition, such reactomics analysis can also be used to generate *in vivo* metabolic pathways composed of connections among metabolites based on high-frequency PMDs. As shown in Figure 3, this form of PMD-based reactomics analysis provides an untargeted method of evaluating *in vivo* SPME studies at the reaction level, which is helpful in discovering new pathways of pollution or influenced endogenous compounds.

## 3 Application of *In Vivo* SPME

In recent years, *in vivo* SPME has been employed in numerous studies to analyze environmental contaminants in complex matrices with results proving its ability to provide useful information about the dynamics of the studied ecosystems.^23,24^ Chemicals such as persistent organic pollutants (POPs), endocrine-disrupting compounds (EDCs), pesticides, PPCPs, disinfection byproducts (DBPs), and heavy metals can be released into environmental matrices such as soil, air, water, and sediments. As a result, plants, animals, and humans may be exposed to these compounds, either through direct contact with environmental matrices or via the food chain. The aforementioned emerging contaminants can accumulate and exhibit toxicity in living organisms, particularly with respect to their influence on different cellular processes at the genomic, proteomic, and metabolomic levels. As such, it is critical to investigate or monitor the behavior, levels, and distribution of these compounds in living systems. Fortunately, tools such as *in vivo* SPME are capable of extracting such exogenous compounds quickly and efficiently, and the development of a commercially available SPME fiber for *in vivo* studies has led to it becoming the preferred technology in an increasing number of environmental studies. Furthermore, *in vivo* SPME has been introduced as a novel technology for simultaneously extracting a wide range of small molecules directly from living systems, which has been key in developing a more robust understanding of how exogenous substances impact the functioning of living organisms. The findings of various metabolomic studies have confirmed the feasibility of using SPME devices to extract and stabilize endogenous molecules—especially highly reactive metabolites or intermediates of biochemical processes—directly from living systems. Table 1 summarizes the application of *in vivo* SPME in environmental science over the past 5 years.

**Table 1:** Application of *In Vivo* SPME in Environmental Science during the Past 5 Years (2016–2021)

analytes	objects	fiber	instrument	extraction mode	ref
alkaloids and metabolome	amazonian plants	custom-made fiber (PAN with either RP-amide or HS-F5 silica particles)	LC-MS	direct immersion into bark or fruit, 30/120 min extraction, 23–50 min desorption	(68)
organochlorine and organophosphorus pesticides	malabar spinach	custom-made PDMS fiber	GC-MS	direct immersion into different organs, 20 min extraction	(69)
carbamazepine and ibuprofen	malabar spinach	C18-fiber	LC-MS/MS	direct immersion into foliage, 45 min extraction, 15 min desorption	(70)
organophosphorus pesticides	cabbage and aloe	custom-made PDMS fiber	GC-MS and LC-MS/MS	direct immersion into foliage, 20 min extraction, 60 min desorption	(71)
neonicotinoids	lettuce and soybean seedlings	custom-made biocompatible fiber (PAN with DMF and PPVP particles)	LC-MS/MS	direct immersion into foliage, 20 min extraction, 2–60 min desorption	(72)
pharmaceuticals and phytohormones	malabar spinach	C18 fiber	LC-MS/MS	direct immersion into stems, 30 min extraction, 10 min desorption	(73)
metabolome	apple	DVB/CAR/PDMS fiber	GCxGC-ToFMS	direct immersion into fruit, 60 min extraction	(74)
carbamate and metabolome	Chinese cabbage plants	polyaminal fiber and three commercial (DVB/CAR/PDMS, PA, and PDMS) fibers	UPLC-FTICR-MS and GC-QToF	direct immersion into foliage, 20 min extraction, 20 min desorption	(75)
metabolome and steroid hormones	white sucker	PAN-C18 TF SPME blade	LC-HRMS and LC-MS/MS	direct immersion into muscle, 20 min extraction, 60 min desorption	(22, 76, 77)
tetrodotoxin	pufferfish	custom-made electrospun PS@PDA-GA fibers	LC-MS/MS	direct immersion into muscle, 45 min extraction, 45 min desorption	(78)
pharmaceuticals	tilapia	custom-made CNT@PPY@pNE fiber	LC-MS/MS	direct immersion into muscle, 1 min extraction, 30 min desorption	(43)
luteolin and metabolites	rat	custom-made MIP SPME fiber	LC-MS/MS	direct immersion into liver, 10 min extraction	(79)
metabolome	rat	commercial C18 and mix-mode (MM) fiber	LC-HRMS	direct immersion into brain, 30 min extraction	(80)
oxylipins	rat	commercial C18 and mix-mode (MM) fiber	LC-MS/MS	direct immersion into brain, 15 min extraction, 60 min desorption	(81)
metabolome	rainbow trout	commercial C18 and mix-mode (MM) fiber	LC-HRMS	direct immersion into muscle, 20 min extraction, 90 min desorption	(82)
PAHs	tilapias	custom-made sheathed MOF fiber	GC-MS	direct immersion into muscle, 30 min extraction	(83)
metabolome	rainbow trout	commercial C18 and mix-mode (MM) fiber	LC-HRMS	direct immersion into muscle, 20 min extraction, 90 min extraction	(60)

### 3.1 *In Vivo* SPME in Plant Analysis

Plants are constantly exposed to various environmental pollutants via the air, water, soil, and sediments. Initially, SPME was introduced as a HS-SPME mode in plant-related research, mainly to extract flavors released by the plants. However, *in vivo* SPME is now usually implemented to track the absorption, transmission, and distribution behaviors of certain exogenous compounds and their metabolites or for the analysis of metabolic pathways (untargeted metabolomics) affected by environmental contaminants. DI-SPME has also been used to investigate the distribution of pharmaceuticals, phytohormones, organophosphorus pesticides, and organochlorine in living plants, with extractions being performed by placing the SPME fibers inside prepunched holes on the foliage.^69,71,73^ Furthermore, several environmental properties, such as distribution concentration factor (DCF), have been calculated to improve our understanding of molecular behavior in living plant sap. In addition, PMDS-coated SPME fibers have been implemented to reduce the matrix fouling during *in vivo* sampling of fat rich matrices, such as avocado.^84^ In another recent study, researchers developed a water-swelling sampling probe to detect neonicotinoids in plants. This fiber decreased the limits of detection for neonicotinoids by introducing a water-swelling structure, thus providing better performance compared to commercially available *in vivo* SPME fibers.^72^

The application of *in vivo* SPME in plant metabolomics is another research trend in environmental studies. Musteata *et al.* evaluated the performance of *in vivo* SPME in Amazonian plants, with results showing that it was able to detect a number of unique compounds.^68^ Risticevic *et al.* utilized *in vivo* SPME and two-dimensional gas chromatography-time-of-flight mass spectrometry (GCxGC-ToFMS) to investigate changes in the “Honeycrisp” apple metabolome profile during maturation.^74^ Their findings showed that several metabolites and chemical classes were upregulated during ripening and that the *in vivo* SPME device was able to successfully extract and detect amaryllidaceae alkaloids as a bioactive metabolite in the analyzed apples. Such compounds have not been previously reported due to changes in metabolite composition during sample preparation (*e.g.*, via the induction of enzymatic degradation and oxidation processes).

DI-SPME is becoming the preferred mode of extraction for *in vivo* environmental studies on plants, tissues, and organs. This approach is especially valuable in metabolomics studies that aim to evaluate the influence of exposure to contaminants beyond their distribution within plants. Nevertheless, new coatings or device geometries are still needed to improve SPME’s overall performance in plant metabolomics, as is the development of novel methodologies that account for the complex matrix effects related to the sampling of living plants.

### 3.2 *In Vivo* SPME in Animal Studies

Unlike plant studies, where the living organism is largely stationary, *in vivo* animal studies are more challenging, particularly with respect to performing extractions. Typically, animal studies are based on the withdrawal of biosamples, such as blood or tissue biopsy, which introduces extra influences that may be harmful to the organism. As a minimally invasive sampling technique, *in vivo* SPME has been applied to monitor the distribution and metabolic processes of certain exogenous (*e.g.*, environmental pollutants) and endogenous compounds (*e.g.*, metabolites) in living animals.^4^ Similar to *in vivo* plant studies, SPME devices used in animal studies must be designed to ensure that the developed method is easy, fast, and feasible for sampling. For HS-SPME, a regular SPME device can be used to extract analytes from collected biofluids. However, *in vivo* tissue or blood sampling—which is where DI-SPME is usually applied—requires a device with low invasiveness, such as the use of an SPME syringe to deliver the SPME fiber into the living system. Notably, the needle of the SPME syringe features a recession notch that is coated with the extraction phase, which allows the delivery device and extraction device to be integrated as one.^31^ Finally, TF-SPME blades can also be used in animal studies, as they offer enhanced sensitivity due to their larger surface area compared to SPME fibers.^22^

*In vivo* SPME animal studies can be divided into two categories: laboratory studies and field (on-site) sampling. Conventional sampling methods can be damaging to living tissue, such as brain tissue; however, the use of biocompatible SPME fibers can minimize the negative effects of sampling and allow researchers to monitor dynamic molecular-level changes in response to certain stimuli or exposures in such sensitive tissue. SPME was initially applied in animals to monitor the pharmacokinetics of toluene in the brains of free-moving mice in order to examine the neural system damage caused by exposure to this compound.^85^ The results revealed a peak concentration of toluene in the hippocampus within 30 min and depletion after 90 min. In another study, an in-depth profiling of 52 oxylipins, which at very low concentrations are lipid mediators of important brain processes, was also carried out *in vivo* by inserting SPME fibers into the brain of freely moving rats.^81^ In another study, deep brain stimulation processes were monitored in rats to track metabolite-level alterations, with findings revealing significant changes in the amino acid, citrulline, as well as in various phospho- and glycosphingolipids.^80^ In addition, *in vivo* sampling of rat liver with custom-made MIP-SPME fibers was successfully applied to monitor luteolin and its metabolites.^79^

Other exposure studies monitoring emerging environmental pollution have been conducted on-site with living aquatic animals. SPME’s ability to extract a wide range of metabolites has also been exploited to analyze the composition of small molecules present in the sponge holobiont.^86^ In this study, different sections of sponges were sampled, and the SPME devices were successfully used to isolate different signaling molecules and organic pollutants, such as monocyclic aromatic hydrocarbons (MAHs) and polycyclic aromatic hydrocarbons (PAHs), among others absorbed from the surrounding water that may accumulate in the sponge and affect its metabolism.

Living fish are commonly used as an *in vivo* animal model to study the exposure effects of pollutants present in water. For this purpose, *in vivo* SPME technology has been applied in EWAS in combination with metabolomics studies in order to track the behavior of certain toxins and monitor biochemical responses after exposure to those factors, mainly in an aquatic environment. Several quantitative *in vivo* SPME analyses of a wide range of pharmaceuticals showed that their concentrations in fish are related to exposure concentrations in effluents. *In vivo* SPME has also been used in wild fish to monitor emerging contaminants in natural fish habitats.^87^ Roszkowska *et al.* employed a case-control *in vivo* SPME experimental design to analyze the changes in the fish muscle metabolome following exposure to benzo[*a*]pyrene. Their findings revealed that the levels of selected amino acids, lipids, and components of osmotic regulation changed in fish muscles after exposure to this toxin.^82^ Moreover, a new SPME device with electrosorption enhancement—namely, a novel, custom-made CNT@PPY@pNE fiber—was employed to monitor ionized acidic pharmaceuticals in fish.^43^ The results of this study showed that the new electrosorption-enhanced fiber was able to provide ultrafast sampling (1 min) and continuous monitoring.

*In vivo* SPME can also be applied to monitor how environmental pollutants impact the functioning of living systems in animals. In a recently published work, the metabolites and potential contaminants of 60 white suckers (*Catastomus commersonii*) in the oil sands development region and outside the deposit region (pulp and paper mill discharge region) were assessed.^4^ To this end, SPME probes were placed in the dorsal-epaxial muscles to facilitate the extraction of various organic pollutants, such as aliphatic and aromatic hydrocarbons, pesticides, PPCPs, and petroleum-related compounds. At the same time, endogenous metabolites such as eicosanoids, linoleic acids, and fat-soluble vitamins were also extracted by the SPME device, revealing significant changes in the biochemical profiles of the components of the white suckers’ skeletal muscle tissue. Exposome studies, along with the metabolomic profiling of endogenous tissue components, enable multifactorial explorations of the cause–effect relationship between exogenous and endogenous molecules in living organisms. This is due to the fact that *in vivo* SPME extracts both endogenous and exogenous compounds, which makes it possible to simultaneously monitor the composition and effects of these pollutants, including their toxicity on the cellular metabolome of living animals.

### 3.3 *In Vivo* SPME in Human Studies

Unlike *in vivo* sampling of plants and animals, *in vivo* studies with humans require even more careful evaluation of the analytical workflow, particularly with respect to extractions. HS-SPME has been used as a diagnostic tool to detect ethanol, acetone, and isoprene in human breath samples.^88,89^ In addition, needle-trap device (NTD) technology coupled with thermal-desorption photoionization time-of-flight mass spectrometry (TD-PI-TOFMS) has also been used for *in vivo* breath sampling of smokers and nonsmokers; the results of this study showed that the device was able to detect xenobiotic substances such as benzene, toluene, styrene, and ethylbenzene in the breath samples collected from the smokers.^90^ Traditional methods for breath or odor analysis need to use a Tedlar bag,^91^ and the sampling process is usually separated with extraction. However, SPME and related techniques can perform sampling and extraction at the same time with a portable design

Saliva is another complex human matrix that can be used for *in vivo* SPME. A review of the literature and an open-source saliva-metabolome database revealed the existence of at least 14 metabolic pathways in the human saliva exposome, including amino acid metabolism, TCA cycles, gluconeogenesis, glutathione metabolism, pantothenate and CoA biosynthesis, and butanoate metabolism. Mixed-mode SPME fibers have also been used to extract metabolites from saliva,^92^ and, more recently, researchers have attempted to perform extractions from saliva via TFME.^76^ To this end, a TFME device was directly placed in the mouth of human subjects for a 5 min extraction, followed by analysis. The analysis showed that TFME enabled the quantification of 49 prohibited substances and provided limits of quantification (LOQs) ranging between 0.004 and 0.98 ng/mL.^93^ Other extraction methods such as LLE can also be applied for exhaustive extraction of saliva samples,^94^ while *in vivo* SPME can capture the free concentration of certain compounds as it extracts via negligible depletion.

Skin odor may also contain information about environmental exposures and endogenous metabolites. HS-SPME has been applied for *in vivo* skin analysis of human scent profiles,^95^ and TFME membranes could be applied directly on the skin to detect semi- and low-volatility compounds *in vivo*.^96^ In addition, *in vivo* SPME has been used to monitor the delivery and impact of doxorubicin on the profile and composition of metabolites in human lungs during the chemoperfusion process.^97^ This approach can also be implemented to evaluate the delivery of xenobiotics from the environment and their impact on the human metabolome. In another study, *in vivo* SPME fibers were used to extract metabolites from 33 fresh and 87 frozen human muscle samples.^98^ The results of these assays showed a shift from the utilization of carbohydrates to the use of lipids for energy production in malignant hyperthermia susceptible individuals.

Due to multiple ethical and legal regulations, the use of SPME devices for the real-time monitoring of exogenous and endogenous compounds in living human systems *in vivo* remains a challenge. However, wearable devices, such as necklaces or pins, may be a promising option for the dynamic monitoring of exposures. Indeed, Smith *et al.* were able to successfully monitor ketamine using a necklace that had been outfitted with an SPME device.^99^ Furthermore, it may be possible to create wearable *in vivo* sampling devices by coating the surface of the devices with biocompatible materials or even using 3D printing to customize the design of such SPME devices.^100^ Such innovations would enable the use of *in vivo* SPME to collect the environmental information for a person, which would in turn allow the construction of a personalized exposure history.^101^

## 4 Conclusions and Future Perspectives

The development of new biocompatible coatings and SPME device geometries has enabled the use of *in vivo* SPME in various environmental studies focusing on the living system of plants, animals, and humans. In addition to providing comparable performance to traditional extraction methods, SPME offers a miniaturized, minimally invasive extraction method that can provide accurate and reliable results in *in vivo* applications aimed at tracking the biological fate of specific exogenous compounds and their metabolites as well as enabling the direct evaluation of metabolic profile changes in the living organs/tissues being studied.

At the current stage, *in vivo* SPME technology still suffers from some limitations. For instance, specific in vivo SPME fibers used for the method development are homemade, which limits its wider availability; however, several works have detailed steps to prepare *in vivo* SPME devices^31,41^ and also some of them have been commercialized as shown in Table 1. Another limitation is the simultaneous calibration of compounds with different physicochemical properties that are extracted from living organism. Specifically, when the standards are not available, qualitative analysis and semiquantitative analysis might be the options for *in vivo* studies. Those limitations share similar scenarios for metabolomics studies and can be improved with the development of other disciplines in this area, such MS-based instrumentation or data analysis. Moreover, method development of *in vivo* SPME is still focused on specific compound(s) or animal/plant models, and rarely applications can be performed on human samples mainly due to ethical issues and law regulations.

In the future, more exploratory and longitudinal studies tracking the fate of environmental pollutants and their impact on biochemical profiles of living organisms should be conducted. At present, most studies consist of laboratory-based evaluations of sampling methods; thus, more field studies are needed to identify other unique features of *in vivo* SPME techniques in environmental monitoring. Laboratory studies examining the effects of toxicant(s) usually provide important information about the fate and impact of single contaminants on living systems, but they may not comprehensively address concerns regarding the exposome’s total impact on the functioning of organisms. In this respect, nontargeted analysis would also be helpful in identifying novel biomarkers of exposure, and, when conducted via *in vivo* SPME, they could extend our chemical knowledge regarding active or short-lived environmental components. In addition, the lack of an active compounds database for environmental studies continues to contribute to a bottleneck in data analysis. However, approaches such as reactomics might allow researchers to skip the annotation of the identified features in untargeted toxicological and metabolomics studies. The incorporation of multidisciplinary knowledge can contribute to the success of *in vivo* SPME sampling projects, as it can encourage the integration of the most advanced techniques in order to solve complex environmental scientific problems. In the future, such an interdisciplinary approach could provide crucial information that will enable a better understanding of the persistence and effects of pollutants in the environment.
